# The stability of soil aggregates in sweet cherry (*Prunus avium* L.) orchards of different ages and varieties

**DOI:** 10.1016/j.heliyon.2025.e42189

**Published:** 2025-01-22

**Authors:** Muhao Chen, Shu Feng, Jun Wang, Mingyu Gao, Min Liu, Kaibo Wang, Zhou-ping Shangguan, Yongwang Zhang

**Affiliations:** a Shaanxi Key Laboratory of Chinese Jujube, Yan’an University, Yan’an, Shaanxi, 716000, China; b State Key Laboratory of Soil Erosion and Dryland Farming on the Loess Plateau, Northwest A&F University, Yangling, 712100, Shaanxi, China; c Institute of Earth Environment, Chinese Academy of Sciences, Xi’an, Shaanxi, 710061, China; d Shaanxi Engineering & Technological Research Center for Conversation & Utilization of Regional Biological Resources, Yan’an, 716000, China

**Keywords:** Sweet cherry varieties, Soil aggregate stability, Soil quality

## Abstract

In recent years, Sweet Cherry (*Prunus avium* L.) have been in high demand and planted in large quantities due to their nutritional value and appealing organoleptic properties. The management mode and species characteristics in the tillage process lead to a decrease in soil quality, and the stability of soil aggregates and decrease in soil nutrients indicate this. However, the effects of different sweet cherry varieties and increasing planting ages on soil quality remain unknown. In this study, soil samples were quantitatively analyzed at different soil depths (0–20 cm, 20–40 cm, 40–60 cm) in cheery orchards of different varieties and ages. The results demonstrated that the particle size content of soil aggregates differed among the varieties of sweet cherry in different soil layers. The mechanical stability of soil aggregates was found to be the lowest in Jimei cherry orchard, where the mass ratio of aggregates with particle sizes exceeding 0.25 mm (R > 0.25) was below the highest 20.99 %, geometric mean diameter (GMD) was below 22.52 %, and mean weight diameter (MWD) was below 17.46 %. In contrast, lower ages demonstrated superior performance in aggregate water stability. The stability of soil aggregates was found to be affected by sweet cherry cultivation, with changes observed in the content of SOC and TN in the surface soil. Principal component analysis indicated that soil quality deteriorated increasingly with ages, while pass-through analysis demonstrated that ages and soil aggregate stability were key factors influencing soil quality. In conclusion, in addition to economic benefits, soil quality should also be protected. This study can help to improve the scientific theoretical basis for the introduction of sweet cherry planting on the Loess Plateau and the management of soil quality.

## 1 Introduction

Sweet cherry (*Prunus avium* L.) is a high-value economic fruit crop [1], favored by consumers for its good organoleptic properties and health benefits. It is one of the most important and popular fruit varieties in temperate climate zones [2,3]. The majority of sweet cherry cultivation occurs in Northeast China and North China. By 2020, the total area dedicated to cherry cultivation in China had reached approximately 1.8 × 10^5^ ha, with an annual yield of over 1 × 10^6^ t. This development signifies China's emergence as a major producer in the global cherry market [4]. At present, a significant proportion of studies on sweet cherry have focused on the micro level, employing genomic analyses to elucidate the response and hormonal regulation of sweet cherry to unfavorable factors such as drought and water stress [5,6], as well as the prevention and control of bacterial strains of infection [7,8]. Moreover, the study of the photosynthetic characteristics of fruit trees in different environments is a pivotal area of research. Photosynthesis exerts a pivotal influence on the growth and development of fruit trees and fruit formation, acting as the primary source of carbon throughout the life cycle [9,10]. The state of soil aggregates is a key determinant of soil health, and a variety of factors related to the nature of the soil influence this state. Fruit tree cultivation is a common practice; therefore, it is important to investigate the main factors related to the influence of soil aggregates [11]. However, the differences in the effects of different varieties of sweet cherries on soil aggregate stability remain poorly understood.

Soil aggregates represent fundamental units of soil structure and serve as crucial storage and conversion sites for soil nutrients [12]. The soil structure exerts a profound influence on gas exchange, nutrient cycling, and water movement within the soil [13]. Furthermore, soil aggregates exert a protective physical effect on soil organic carbon, with the pore structure within them facilitating organic carbon sequestration. Studies have demonstrated a correlation between soil aggregates content and the content of organic carbon and nitrogen in the soil [14]. Some studies have demonstrated a significant positive correlation between the content of soil macroaggregates and the content of soil organic carbon and total nitrogen [15]. Moreover, it has been demonstrated that soil macroaggregates have a beneficial effect on the accumulation of microbial content. This suggests that soil aggregates are of considerable importance in driving the development of soil structures and changes in ecological functions [16]. Because the stability of soil aggregates is a key factor in improving soil productivity, promoting plant growth, preventing soil degradation, and enhancing erosion resistance, the stability index of soil aggregates is used as an index factor to describe the stability of the soil structure. The disintegration of soil aggregates is a key process in soil erosion, and soil aggregate stability can be used as an important indicator of soil erosion resistance [17]. In order to express soil aggregate stability, two distinct sieving methods were employed: dry sieving and wet sieving. These methods simulate the effects of mechanical tillage and precipitation erosion on the soil. Furthermore, there is a debate as to whether a uniform particle size ratio for dry sieving before wet sieving will affect the results of the latter [18]. It is widely acknowledged that the indicator of water-stable aggregates with a diameter greater than 0.25 mm represents a pivotal marker for the assessment of soil fertility, in conjunction with other key indicators such as mean weight diameter (MWD), geometric mean diameter (GMD), and percentage of aggregate destruction (PAD) [19].

The development of the fruit tree economy has led to the emergence of a number of problematic agricultural management practices. These include the excessive use of fertilizers and herbicides, as well as inappropriate watering and mulching techniques. These practices have the potential to cause a number of ecological issues, including soil degradation and reduction in soil erosion resistance [20]. The maintenance of soil fertility and nutrient content has become a significant area of research in 2024. Yang et al. showed that the stability of soil aggregates and the content of soil organic matter in 4–10year-old apple orchards planted in the Loess Plateau were significantly lower than those observed in ecological plantations. Consequently, the planting of apples should be approached with caution facilitate the sustainable development of the area, particularly in arid regions [21]. In apple orchards on the Weibei Arid Plateau, the stability of soil aggregates was a significant factor in soil compaction [22]. The use of different soil management systems, such as ground cover, has been found to be effective in maintaining the stability of soil aggregates. The study also examined the water stability and carbon sequestration capacity of soil aggregates in vineyard soils, as well as the effect of construction residues on soil aggregate stability and carbon content [23]. In addition, the impact of economic fruit forest planting on soil aggregate stability in subtropical hilly areas is worth considering. The majority of five-year-old peach, pear, citrus, and kiwifruit forests demonstrate mechanical stability. However, it has been observed that soil stability tends to deteriorate with increasing soil depth. The economic fruit forest type has been observed to improve the stability of the soil structure, primarily by affecting the content of large-grained agglomerates. This effect appears to be most pronounced at shallow depths, with the stability of soil aggregates and the decrease in stability with increasing soil depth being less pronounced [24].

The cultivation of economic fruit trees confers significant benefits, yet it also affects the local soil structure and stability. However there is a paucity of systematic research on the effect of sweet cherry cultivation on soil aggregate stability in the Loess Plateau region. Therefore, the present study selected sweet cherries of different planting years, varieties and soil depths (0–20 cm, 20–40 cm and 40–60 cm) as research objects. The following hypotheses were formulated for this study: (1) The stability of soil aggregates and soil quality decreased with increasing sweet cherry planting years; (2) Different varieties of sweet cherries with the same planting years had different effects on soil quality, among which Hongdeng maintained the soil quality to the greatest extent.; (3) SOC and TN in the topsoil are key factors affecting the stability of soil aggregates, thus affecting soil quality. The objective of this study is to elucidate the differences in soil aggregate stability between different varieties of sweet cherries and the influencing factors, we aim to improve the scientific theoretical basis for the selection of suitable cultivars of sweet cherries in the Loess Plateau region and the sustainable management methods employed in the field.

## 2 Materials and methods

### 2.1 Study area

**Fig. 1:**
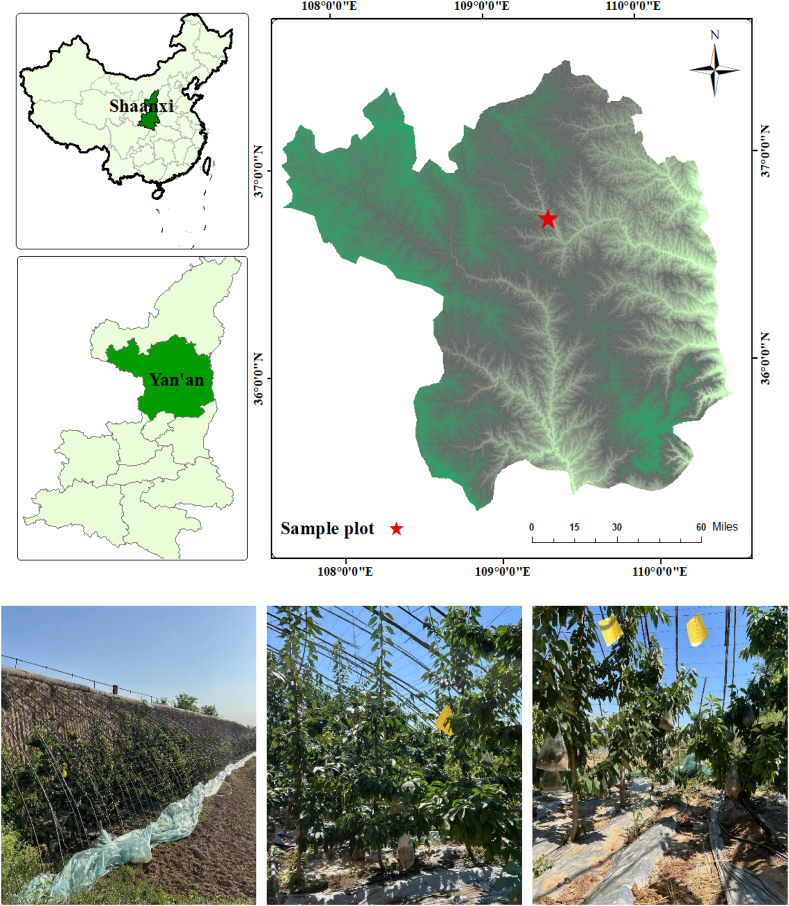
Location of the Loess Plateau and the study plot.

The experimental sample plots were set up in Panpo Mountain, Ansai District, Yan'an City, northern Shaanxi Province (36° 75′ N,109° 30′ E) (Fig. 1). The region falls within the typical hilly and gully area of the northern Shaanxi Loess Plateau, situated at an altitude of approximately 1,700m above sea level. The area exhibits a mid-temperate continental semi-arid monsoon climate, characterized by four seasons of varying lengths, with distinct wet and dry periods. The mean yearly temperature is 8.8 °C, with extreme maximum and minimum temperatures recorded as 36.8 °C and −23.6 °C, respectively. The average annual precipitation amounts to 505.3 mm, with the greatest and the least precipitation sums reaching 645 mm and 296.6 mm, correspondingly. The annual sunshine hours accumulate to 2395.6 h, of which 54 % is sunshine. The frost-free period spans 157 days each year.

The experimental cherries were subjected to a uniform water and fertilizer management pattern, with the same planting spacing and trellis wall orientation, irrespective of planting age (Fig. 1). The sweet cherry greenhouse was covered with plastic sheets and insulated with quilts. De-sheeting was performed in late May, new sheeting was applied and insulated with quilts in September, and quilts were put up at night in January. The greenhouse is situated facing east-west. Cherry trees are typically planted in rows at intervals of 1.5 m × 3.0 m. During the cherry tree budding phase, there is widespread irrigation and application of mulch while laying down water pipes. Prior to the flowering phase, extensive watering was performed, followed by drip irrigation through water pipes a week later. Irrigation of the trees during the late flowering phase and once before the color change phase. Do not irrigate during the color change phase. After the color change phase, soil color and dryness were manually monitored with drip irrigation.

The test cherries were utilized with varied growth years and diverse varieties across thirteen years of growth: Hongdeng (Cerasus pseudocerasus, Dalian Agricultural Science Research Institute in 1963 to Napoleon x Huangyu cross-breeding, named in 1973), Tieton (*Prunus avium* ‘Mei Zao’, Developed by Washington State University in the US, this cultivar results from Stella x EarlyBudm cross-breeding), Jimei (It is primarily cultivated in southern Shaanxi, including the Shaanxi Weibei and Guanzhong regions), Raninier (Named after Mount Rainier in Washington State, it was introduced to China in 1989 by the Dalian Institute of Agricultural Science and promoted in the Dalian area), Summit (A moderately late-ripening cultivar originated by the Canadian Summerland Agricultural Research Institute and brought forth by the Yantai Fruit Tree Research Institute in 1988), four years of age: Jimei(4aJimei), Tieton (4aTieton), two years of age: Tieton (2aTieton).

### 2.2 Soil sampling

In order to investigate the impact of different planting years and varieties of sweet cherry on soil organic carbon, total nitrogen, quick-acting phosphorus, and quick-acting potassium, a soil auger was used to take three points in the soil depth of 0–20 cm, 20–40 cm, and 40–60 cm. These samples were then analyzed to determine the aforementioned indices.To select three additional sample points and utilize a soil shovel to excavate the soil in situ into a hard plastic box. To transport the soil samples back to the laboratory, where they will be subjected to further processing. This involves the removal of gravel, plant and animal residues, and the drying of the samples at room temperature. The soil will then be broken into small pieces of approximately 1 cm in diameter to determine the soil aggregation stability indicators. Subsequently, a ring knife with a volume of 100 cm^3^ was employed to collect soil at the corresponding soil depth for the determination of soil bulk density, porosity and water content.

### 2.3 Soil aggregate separation

The dry sieving method was employed to analyze the undisturbed air-dried soil. The soil was placed on a sieve mesh, which was arranged in order from top to bottom according to the size of the sieve holes (>2, 2-1, 1-0.5, 0.5-0.25, <0.25 mm). The soil was then placed on a shaking table at a speed of 60 times/minute and shaken for 2 min. Subsequently, soil samples were collected and weighed from each sieve mesh level in order to calculate the percentage content of soil aggregates at each particle level, with the objective of determining the mechanical stability index of soil aggregates.

The wet sieve method was employed to ascertain the water stability of soil aggregates, with the percentage content of each particle size obtained by the dry sieve method serving as the basis for the determination. A total of 50 g of mixed soil samples were combined in accordance with the appropriate ratio. The sieve mesh was arranged in order from large to small and placed on the oscillating frame of the wet sieve instrument, ensuring that the upper and lower oscillations were fully immersed in the water. The instrument was operated at a frequency of 30 times/minute, with an amplitude of 3 cm, for a period of 30 min. Once the wet sieving process was complete, the soil particles of each size were gently rinsed into the evaporation dish using deionised water. After evaporation, the soil aggregates of each particle size were weighed and calculated.

### 2.4 Analysis of soil properties

Soil conductivity (Ec) was determined using a conductivity meter; soil pH was determined using a pH meter (1:2.5 soil-water ratio); soil bulk density (BD), soil water content (SWC) and soil porosity (SP) were determined using the ring knife method; soil organic carbon (SOC) was determined using the potassium dichromate method. The total nitrogen (TN) content of the soil was determined using the semi-micro-volume Kjeldahl nitrogen method. The phosphorus (AP) content was determined using the molybdenum antimony colourimetric method. The potassium (AK) content was determined by flame photometric method with ammonium acetate leaching [25].

### 2.5 Soil aggregate and soil quality calculation

Soil aggregate stability is an important diagnostic index of soil aggregates. The mechanical stability of soil aggregates can be determined by the mean weight diameter (MWD; mm), geometric mean diameter (GMD; mm), and the mass ratio of aggregates with particle sizes exceeding 0.25 mm, R > 0.25 %. These three indexes, MWD, GMD and R > 0.25, are comprehensive indexes characterizing the composition of the diameter sizes of soil aggregates. The greater the MWD, GMD and R > 0.25, the more stable the aggregates.

(1) $$MWD=\sum_{i=1}^{n}\left(\bar{x_{i}}w_{i}\right)$$

(2) $$GMD=\exp\left(\sum_{i=1}^{n}W_{i}\;\ln\bar{x_{i}}\right)$$

(3) $$R_{0.25}=m_{0.25}∕m_{t}\times 100\%$$

Where *xi* is the average diameter of any particle size agglomerate (mm); *wi* is the mass of the *i*th particle size agglomerate as a percentage of the total agglomerate (%). Soil aggregate water stability indicators using Water Stable Aggregates (WSA) and Percentage of Aggregate Disruption (PAD) expression, WSA characterization of water stability agglomerate content, the higher the WSA, indicating that the agglomerate water stability is stronger; PAD combined with the wet and dry sieve method to characterize the mechanical stability of the large agglomerates (>0.25 mm) after wet sieve broken into small agglomerates (<0.25 mm) proportion, PAD. The smaller the PAD, the more stable the agglomerates. MWD and GMD after wet sieving can also be used as indicators of the water stability of soil aggregates.

(4) $$WSA=\frac{{WM}_{>0.25}}{M_{T}}$$

(5) $$PAD=\frac{{DM}_{>0.25}-{WM}_{>0.25}}{{DM}_{>0.25}}$$

Where *WM* > 0.25 is the mass of agglomerates (g) for wet sieve >0.25 mm; *DM* > 0.25 is the mass of agglomerates (g) for dry sieve >0.25 mm; *MT* is the total mass of wet sieve agglomerates (g).

Soil quality index (SQI) is a comprehensive reflection of soil properties, through the principal component analysis (PCA) of soil property indicators to select the principal component with eigenvalue greater than 1 and the high factor loading indicators in the principal component whose absolute value reaches 90 % of the maximum factor loading, to obtain the common factor inverse of each indicator and the weight value of each indicator, to standardize the data of the soil indicators and then to use the obtained weights on the soil indices comprehensive evaluation, the higher the SQI value, the better the soil quality.

Redundancy analysis (RDA) was carried out with soil aggregate stability indicators as the response variable and soil physical and chemical properties and the explanatory variables to find the key indicators affecting soil aggregate stability.

### 2.6 Statistical analyses

Statistical analyses were performed using SPSS 19.0 and visualizations were performed using Origin 2022. One-way analysis of variance (ANOVA) was used to test the response of soil aggregate stability indicators and soil physicochemical properties to different varieties of sweet cherry. Pearson correlation analysis was used to evaluate the relationship between the indicators. Soil aggregate stability, nutrients, and properties were determined using structural equation modelling (SEM). The effects of different varieties and different planting years of sweet cherries on soil quality were visualized. ArcMap 10.8 was used to map the experimental sites.

## 3 Results

### 3.1 Composition of soil aggregates

**Fig. 2:**
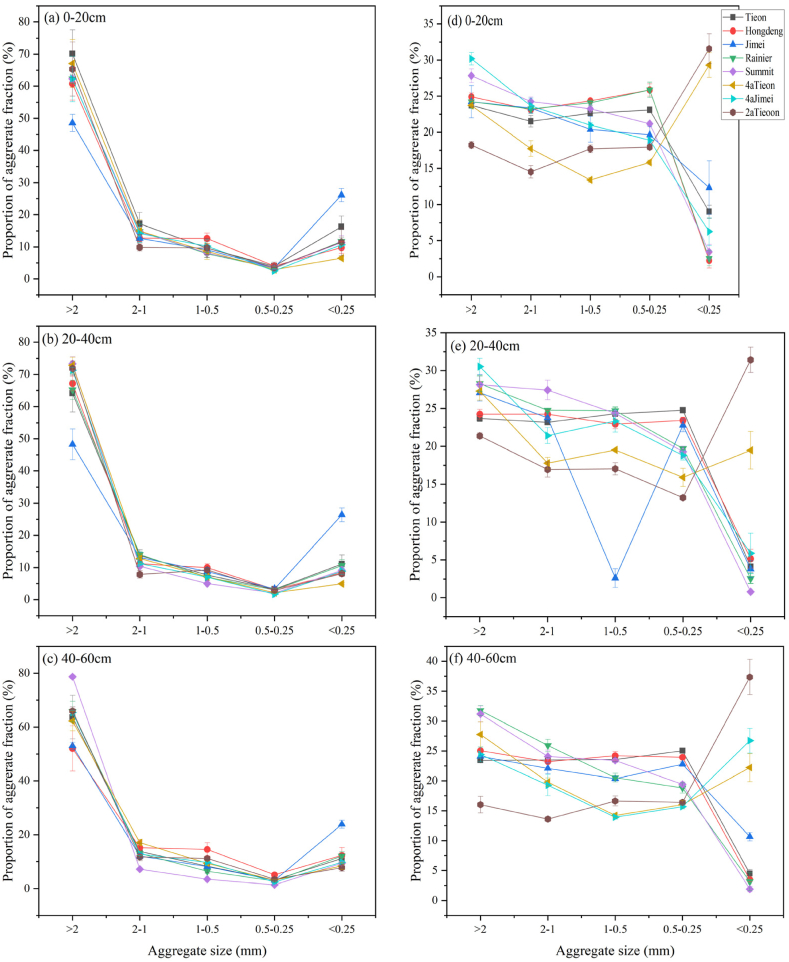
Percentage (%) of each particle size of soil aggregates to the total weight of the soil after dry sieving (a, b, c) or wet sieving (d, e, f) for different varieties of sweet cherry soils.

The percentage content of soil aggregates of different grain sizes after dry sieving of in situ soils from three different soil layers (0–20, 20–40, and 40–60 cm) demonstrated a uniform trend of decreasing and then increasing with smaller grain sizes. Jimei exhibited a lower content of aggregates >2 mm, as well as the highest content of aggregates with grain sizes <0.25 mm at all three soil depths (Fig. 2).

Following wet sieving, the percentage of soil aggregates of different particle sizes for the majority of tree species demonstrated a trend of decreasing, then increasing, and then decreasing at varying soil depths. At soil depths of 0–20 and 20–40 cm, 4a and 2a Tieon exhibited an increasing trend at soil aggregate particle sizes of <0.25 mm. At soil depths of 40–60 cm, all soil aggregates except for 4a and 2a Tieon, 4a Jimei, also demonstrated an increase in the percentage content of <0.25 mm particle size (Fig. 2).

### 3.2 Stability of soil aggregate

**Fig. 3:**
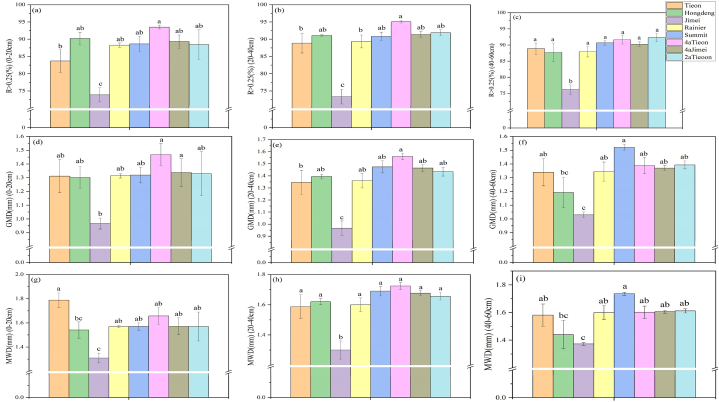
Differences in mechanical stability of soil aggregates after dry sieving of different soil depths of different varieties of sweet cherries, the mechanical stability of soil aggregates can be determined by the mean weight diameter (MWD; mm), geometric mean diameter (GMD; mm), and the mass ratio of aggregates with particle sizes exceeding 0.25 mm (R > 0.25 %), with lower case letters representing significant differences (*P* < 0.05), are shown below.

The values calculated from the percentage content of individual particle sizes of soil aggregates after dry sieving were employed as indices representing the mechanical stability of soil aggregates (R > 0.25, GMD, and MWD). The values of R > 0.25 for Jimei were significantly lower (*P* < 0.05) than those of the other varieties at the three different soil depths, with values smaller than the highest levels of 20.99, 22.52, and 17.46 percent, respectively. In contrast, the species with lower planting years exhibited higher values of R > 0.25 at different soil depths. The GMD and MWD at different soil depths were significantly lower for Jimei than for the other species, as well as for those with fewer planting years. The results demonstrated that the mechanical stability of soil aggregates in Jimei was significantly (*P* < 0.05) lower than that of other tree species at different soil depths. Furthermore, no significant differences in the mechanical stability of soil aggregates were observed among the other tree species (Fig. 3).

**Fig. 4:**
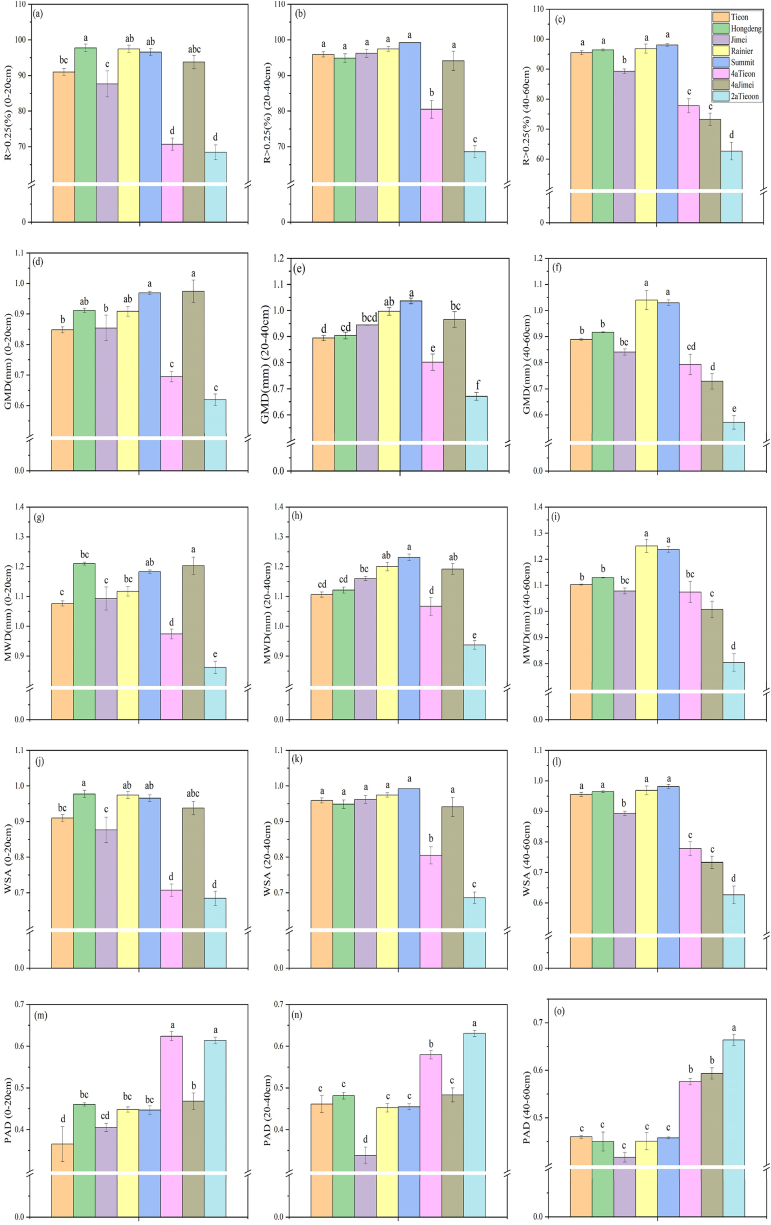
Differences in mechanical stability of soil aggregates after wet sieving of different soil depths of different varieties of sweet cherries. Water Stable Aggregates (WSA) and Percentage of Aggregate Disruption (PAD).

The percentage content of different particle sizes of soil aggregates after wet sieving was used to calculate and express the indicators of water stability of soil aggregates (R > 0.25, GMD, MWD, WSA, and PAD). It was observed that as the PAD increased, the water stability of the aggregates decreased, whereas the other indicators exhibited an inverse relationship, indicating that as the values of these indicators increased, the water stability of the aggregates improved. At different soil depths, the two lower planting years in Tieon exhibited significantly lower indicators of aggregate water stability than the other species at 0–20 cm as well as 20–40 cm. Additionally, 4a Jimei exhibited lower aggregate water stability at 40–60 cm soil depths. Conversely, the different aggregate water stability at various soil depths of the Rainier and Summit indicators performed better (Fig. 4).

### 3.3 Nutrients and properties of soil

**Fig. 5:**
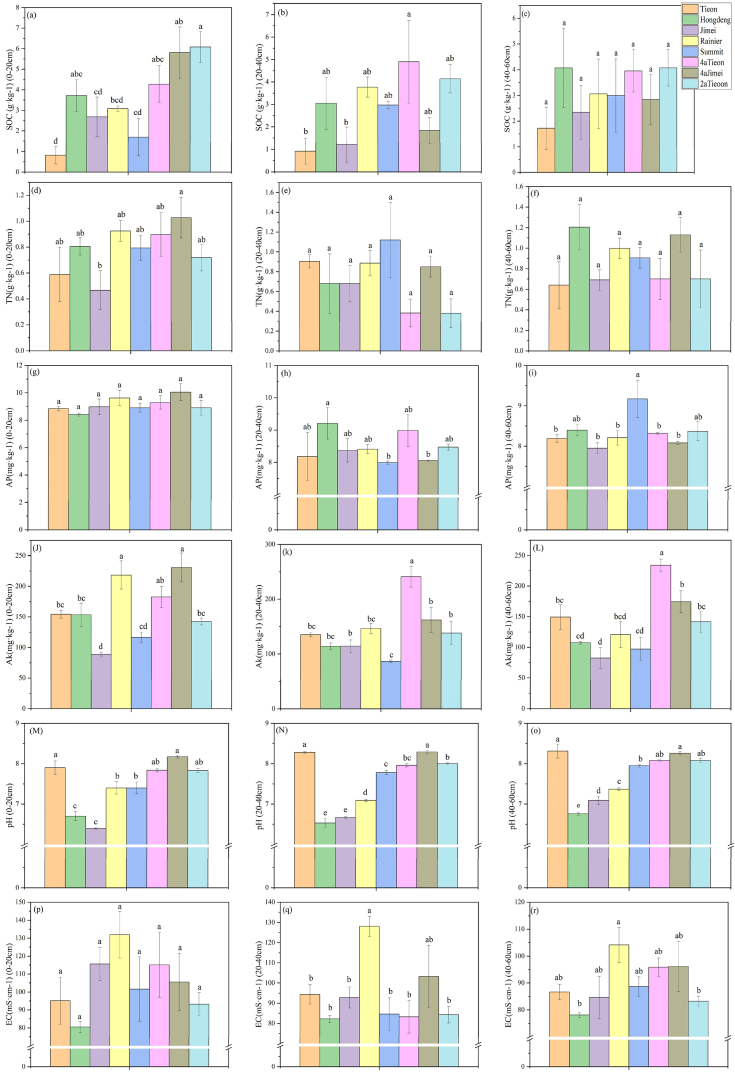
Differences in soil properties of different varieties of sweet cherries at different soil depths.

With regard to the physicochemical properties of different varieties of sweet cherry soils, it was observed that the SWC and SP of Rainier and Summit soils were significantly lower (*P* < 0.05) than those of the other species at different soil depths. In contrast, the BD, Jimei and 2aTieon levels were lower at 0–20 cm and 20–40 cm, while the BD of Summit was significantly higher than that of all other species at 40–60 cm. The Hongdeng and Tieon levels were significantly lower than those of Rainier, Summit and the same species with fewer planting years (Table 1). The pH values of Hongdeng and Jimei were significantly lower than those of the other species at soil depths of 0–20 cm. Furthermore, Rainier exhibited lower pH values than the other species at soil depths of 20–40 cm and 40–60 cm. No significant differences were observed among the individual species for EC at 0–20 cm. However, Rainier exhibited a significantly higher EC than the other species at 20–40 cm. The EC of Rainier at 40–60 cm was significantly higher than that of Hongdeng and 2aTieon (Fig. 5).

**Table 1:** Differences in soil water content (SWC), soil bulk density (BD) and soil porosity (SP) of different varieties of sweet cherry at different soil depths.

Type	Soil depth								
	SWC			BD			SP		
	0–20	20–40	40–60	0–20	20–40	40–60	0–20	20–40	40–60
Tieon	14.38 ± 0.26bc	13.46 ± 0.32d	13.21 ± 0.46b	1.80 ± 0.05a	1.63 ± 0.02c	1.61 ± 0.04bc	40.95 ± 1.05bcd	45.12 ± 1.73a	45.93 ± 1.32 ab
Hongdeng	17.13 ± 0.88a	14.96 ± 0.46cd	13.48 ± 0.79b	1.84 ± 0.02a	1.74 ± 0.02bc	1.52 ± 0.08c	43.15 ± 0.65abc	44.16 ± 1.70a	51.03 ± 4.13a
Jimei	10.39 ± 0.70d	10.46 ± 0.25e	9.77 ± 0.32c	1.65 ± 0.04b	1.82 ± 0.01b	1.78 ± 0.03 ab	42.84 ± 0.84abc	42.90 ± 0.53a	40.43 ± 0.18bcd
Rainier	12.39 ± 0.56cd	10.91 ± 0.47e	10.37 ± 0.37c	1.87 ± 0.03a	1.79 ± 0.05bc	1.86 ± 0.01a	35.39 ± 1.71e	36.54 ± 1.06b	34.86 ± 0.43d
Summit	14.88 ± 1.14 ab	16.01 ± 0.48bc	14.56 ± 0.80b	1.90 ± 0.01a	2.10 ± 0.05a	1.89 ± 0.14a	37.40 ± 1.96de	37.64 ± 1.17b	38.54 ± 1.40cd
4aTieon	16.86 ± 0.15a	17.65 ± 0.59 ab	18.04 ± 0.27a	1.81 ± 0.01a	1.85 ± 0.03b	1.88 ± 0.01a	44.32 ± 1.15 ab	45.28 ± 0.56a	46.54 ± 2.66 ab
4aJimei	16.67 ± 0.98 ab	19.01 ± 0.75a	17.59 ± 0.23a	1.48 ± 0.06c	1.82 ± 0.06b	1.89 ± 0.09a	46.29 ± 1.26a	44.00 ± 0.77a	40.51 ± 2.06bcd
2aTieon	14.35 ± 0.47bc	14.45 ± 0.42cd	14.58 ± 0.24b	1.89 ± 0.06a	1.75 ± 0.10bc	1.80 ± 0.00 ab	39.61 ± 0.46cde	43.88 ± 1.20a	43.39 ± 0.63bc

Note: Values are in the form of Mean ± SE and the sample size n = 3. Different lower-case letters mean significant differences in the same factor at the different varieties of sweet cherry. (*P* < 0.05).

Soil organic carbon (SOC) at different soil depths was found to be higher for species with fewer planting years. However, total nitrogen (TN) did not show significant differences at other soil depths, with the exception of Jimei, which was found to be significantly smaller than the other species in the 0–20 cm soil layer. Additionally, available phosphorus (AP) did not show significant differences in the 0–20 cm soil depth, with Hongdeng being found to have significantly higher AP content than Summit and 4aJimei at 20–40 cm soil depth, and Summit showed a higher level of AP content at 40–60 cm soil depth. Furthermore, available potassium (AK) was found to be higher at 0–20 cm soil depth in Rainier, while all other soil depths showed a higher level of AK content in species with lower planting years (Fig. 5).

### 3.4 Correlations between soil properties and aggregates

**Fig. 6:**
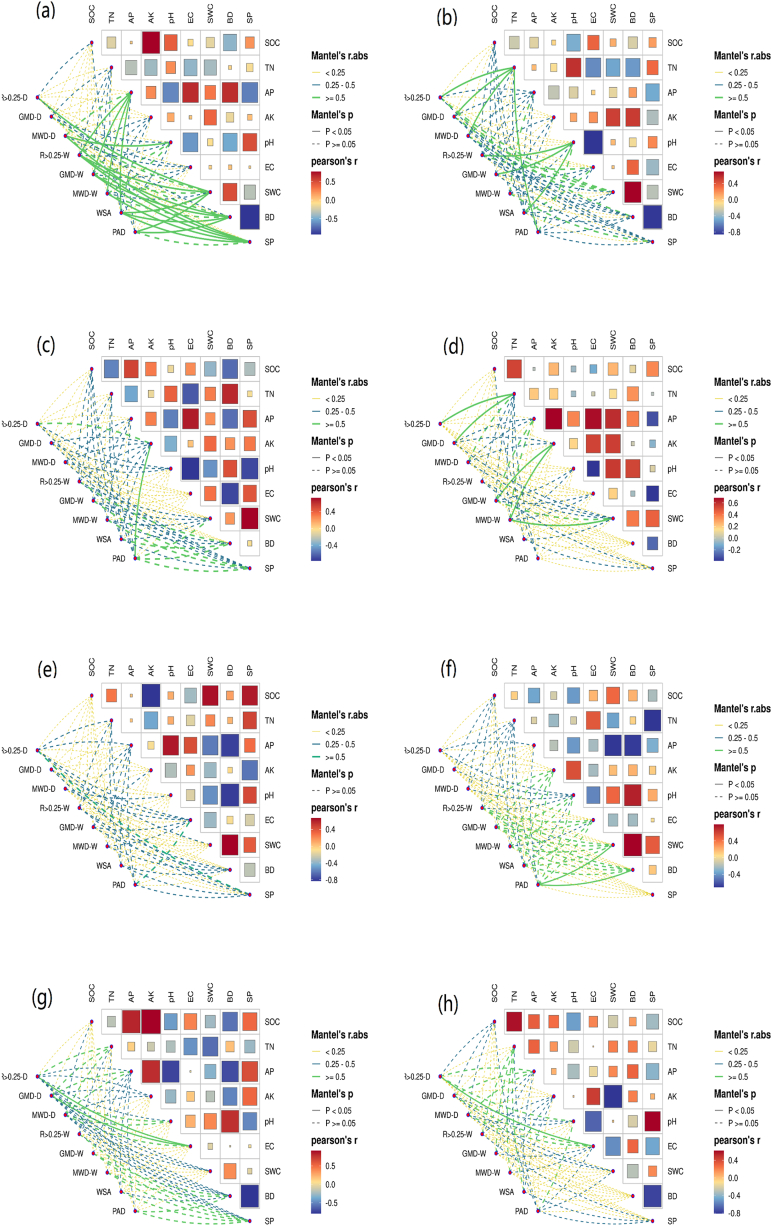
Differences in the stability of soil aggregates and the correlation between soil physico-chemical properties in different varieties of sweet cherry (The alphabetical order is in order: Tieon, Hongdeng, Jimei, Rainier, Summit, 4aTieon, 4aJimei, 2aTieon).

The Mantel test was employed to analyze a range of indicators pertaining to the stability of soil aggregates and properties of different varieties of sweet cherries. Additionally, correlation heat maps were constructed to illustrate the relationships between the soil properties. The analysis revealed that the AP content of Tieon had a significant effect (*P* < 0.05) on the water stability index of soil aggregates, while pH had a significant positive effect on MWD-D, a significant negative effect on BD, and a significant positive effect on SP, which also significantly affected the soil aggregate stability indices. Furthermore, the TN and pH of Hongdeng significantly affected the soil aggregate stability. In addition to the above physicochemical properties, the concentrations of available potassium (AK) and electrical conductivity (EC) were observed to be significant indicators of soil aggregate stability for more species. The negative effects of soil moisture content (SWC), bulk density (BD), and soil pH (SP) on soil organic carbon (SOC), total nitrogen (TN), and available phosphorus (AP) at low planting age gradually diminished or became promoters as planting ages increased. Furthermore, a general promotion effect was observed for SOC, TN, AP, and AK (Fig. 6).

**Fig. 7:**
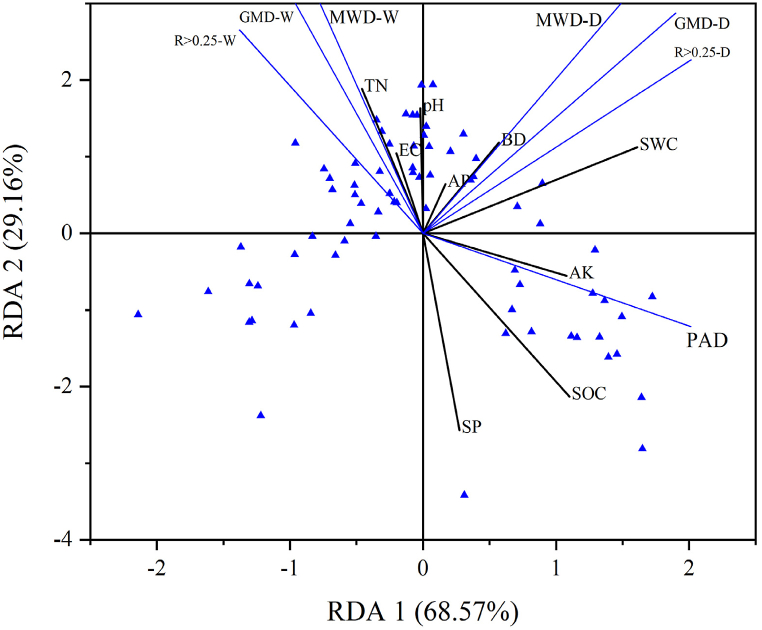
Redundancy analysis of soil aggregate stability indicators and soil physico-chemical properties.

Redundancy analysis (RDA) was employed to categorize distinct varieties of sweet cherries in order to minimize discrepancies in data units and to standardize the data. This was achieved by utilizing indicators of soil aggregate stability as the corresponding variables and soil physical and chemical properties as explanatory variables. The results demonstrated that the first and second principal axes explained 68 % of the variance in aggregate stability. The variance in aggregate stability was explained by 57 % and 29.16 %, respectively. SWC had the greatest positive effect on soil mechanical stability, TN had the greatest positive effect on soil water stability, and SOC was a key factor affecting soil stability (Fig. 7).

### 3.5 Soil quality comparison and driving factors

**Fig. 8:**
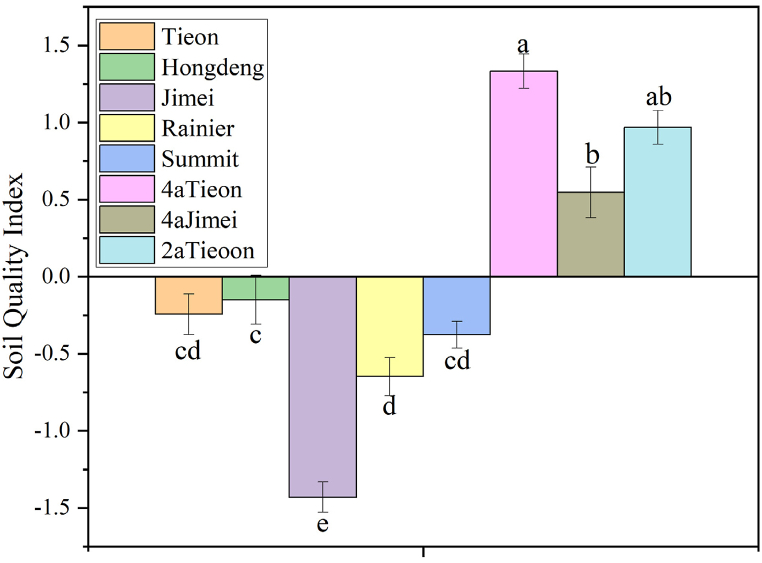
Differences in soil quality indices of different sweet cherry varieties.

Principal component analysis (PCA) was used to analyze the soil aggregate stability indices and soil physicochemical factors of different varieties of sweet cherries. Assuming no loss of variance, the eigenvalues of the first six principal components were greater than 1, and the cumulative variance contribution ratio reached 83. The results indicated that these six principal components could effectively replace the soil aggregate stability indexes and soil physicochemical factors and could be used to evaluate and analysis the characteristics of soil quality (Table 2). The corresponding principal component scores were weighted to obtain a comprehensive evaluation function, which was used to estimate the soil quality index (SQI). The results demonstrated that the larger the composite value, the better is the soil quality. The SQI index of Tieon with fewer planting years was found to be significantly larger than that of the other varieties. The SQI index of the eight sweet cherries of different varieties and planting years were ranked as follows: 4aTieon > 2aTieon > 4aJimei > Hongdeng > Tieon > Summit > Rainier > Jimei (Fig. 8).

**Table 2:** Seventeen indicators of soil quality characteristics of eight cherry samples were analyzed by principal components. The first six principal components were found to effectively replace the other indicators. The loading matrices and variance contributions are presented in the table.

Index	Principal Component					
	1	2	3	4	5	6
R > _0.25_-D	0.590	0.729	0.063	−0.114	0.018	−0.029
GMD-D	0.507	0.809	−0.069	−0.113	0.088	−0.154
MWD-D	0.422	0.792	−0.143	−0.125	0.094	−0.219
R > _0.25_-W	−0.887	0.386	0.154	−0.062	−0.083	−0.033
GMD-W	−0.839	0.490	0.118	−0.07	−0.070	0.013
MWD-W	−0.796	0.506	0.143	−0.006	−0.101	0.003
WSA	−0.887	0.386	0.154	−0.062	−0.083	−0.033
PAD	0.951	0.047	−0.047	0.014	0.072	0.067
SOC	0.488	0.028	0.343	0.316	0.023	0.266
TN	−0.214	0.188	0.085	0.007	0.078	0.859
AP	0.080	0.168	0.491	0.558	0.307	0.033
AK	0.459	0.219	0.411	0.375	−0.391	−0.010
Ph	−0.089	0.166	0.193	−0.081	0.856	−0.030
EC	−0.101	0.132	−0.042	0.793	−0.140	−0.230
SWC	0.563	0.451	00.245	−0.275	−0.291	0.186
BD	0.113	0.338	−0.758	0.112	−0.130	0.250
SP	0.247	−0.294	0.692	−0.480	−0.192	--0.095
Eigenvalue	5.507	3.172	1.800	1.555	1.196	1.048
Variance contribution rate %	32.396	18.659	10.586	9.146	7.034	6.164
Cumulative proportion in ANOVA %	32.396	51.054	61.641	70.787	77.820	83.985

**Fig. 9:**
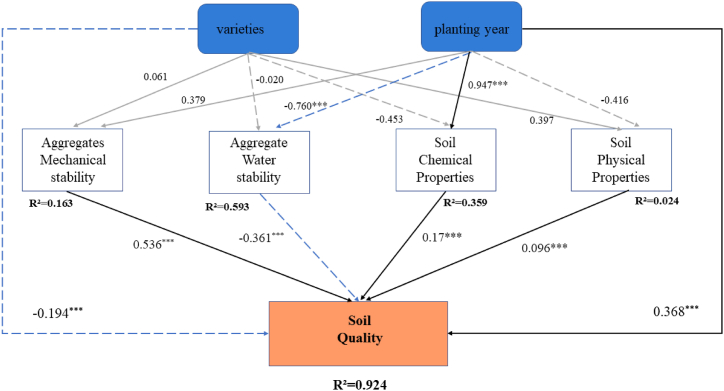
The effects of sweet cherry variety and planting year on soil quality were estimated by means of a through path analysis. Positive effects are indicated by solid lines and negative effects by dashed lines. Black and grey lines are significant and insignificant, respectively. R^2^ denotes the coefficient of the inner model. ∗*P* < 0.05; ∗∗*P* < 0.01; ∗∗∗*P* < 0.001.

Pathway analyses demonstrated that the soil quality was significantly influenced by the variety and planting year of sweet cherry trees. The presence of water-stable soil aggregates had a negative effect on soil quality. In contrast, sweet cherry varieties did not have a significant effect on soil aggregate stability or on physicochemical factors. Furthermore, planting age had a highly significant negative effect on soil aggregate water stability and a highly significant positive effect on soil chemical properties (Fig. 9).

## 4 Discussion

### 4.1 Effects of terracing on composition and stability of soil aggregates

The results demonstrated that the particle size distribution of soil aggregates in different soil layers after dry sieving exhibited a uniform trend, with a relatively large proportion of soil aggregates comprising large aggregates [26]. Furthermore, the mechanical stability of the soil aggregates in most sweet cherries did not exhibit significant differences. However, the mechanical stability index of soil aggregates in Jimei exhibited the poorest performance. Moreover, disparate soil aggregate stabilities were observed even when the planting conditions and years were identical, which may be attributed to the tree species [15]. This discrepancy may be attributed to the characteristics of the tree species, the utilization of fruit/seed mucilage as a natural soil stabilizer, which enhances the stability of soil aggregates, and the decomposition of tree litter and the adhesive effect of root secretions [27,28], which alter the stability of soil aggregates. Microagglomerates can withstand significant mechanical and physicochemical stresses, thereby playing a pivotal role in the mechanical stability of the soil [29].

Following wet sieving of soil samples from different layers, it was observed that the percentage of soil aggregate particle size in sweet cherry trees planted for fewer years exhibited a similar trend. Specifically, there was an increase in the content of aggregates with a diameter of less than 0.25 mm, whilst the index of soil aggregate water stability decreased. This finding lends support to the hypothesis that long-term planting of tree species could enhance the stability of soil aggregates [30].

As the number planting years increases, tree species play an important role in soil and water conservation [31]. This objective is realised through the implementation of strategies aimed at mitigating soil erosion and surface runoff, while concurrently promoting the transformation of microaggregates into macroaggregates. The outcome of this process is enhanced water stability of soil aggregates, improved soil nutrient accumulation, and enhanced soil structure [32].

### 4.2 Effect of physicochemical properties of soils of different varieties of sweet cherries

Soil aggregates have been shown to play a pivotal role in the storage of soil nutrients. The interaction between soil nutrients and the soil aggregate structure has been identified as a crucial factor in this regard [33]. Soil water content (SWC) is regarded as a pivotal factor influencing soil aggregates [34]. SWC influences soil porosity, and, consequently affects the structure and composition of soil aggregates. Differences in SOC and TN between different sweet cherry varieties were primarily observed in the 0–20 cm and 20–40 cm surface soils. The concentrations of SOC and TN were found to be crucial for evaluating the soil quality [35]. The results of the study on the differences in soil SOC and TN were similar to the results of a study on nutrients with significant variations observed in the surface soil layer [36,37]. Soil fast-acting nutrients affect soil aggregate stability by influencing soil microorganisms [38]. The results demonstrated that the different sweet cherry AP and AK contents were higher in the 0–20 cm soil layer, with differences occurring mainly in deeper soils with lower contents.

Soil pH, electrical conductivity (EC), hydraulic conductivity, and porosity are all physical properties that are closely related to soil aggregates. The results of this study demonstrate that an increase in soil porosity and a decrease in soil water content affect the increase in soil water-stable aggregates, which in turn affects the water stability of soil aggregates [39,40]. Furthermore, the pH and EC values were higher in the lower planting years than in the longer planting years, irrespective of the soil horizon.

The physicochemical properties of soil are intimately connected to fertility, and they exert a profound influence on and constraint upon soil microbial activities, soil mineral nutrient conversion, the distribution and replenishment of nutrients [41]. The decomposition of roots and litter during the growth of tree species, as well as the application of fertilizers during planting and management, affects the nutrient content of different soil layers [42]. In particular, the P content is relatively stable, and often influenced by soil type and climate type.

### 4.3 Differences in soil aggregates of different sweet cherry varieties and their influencing factors

The fertilisation measures employed in the context of orchard planting management have been shown to facilitate the accumulation of nutrients in the surface layer of the soil [43,44]. The stability of soil aggregates is influenced by the main impact of the soil nutrients, namely SOC and TN, this is due to the close coupling relationship between C and N, which in turn affects the stability of soil aggregates [45]. The differences in SOC and TN between species may be attributed to the varying accumulation of apoplastic characteristics among tree species, the fall of material to cover the surface of the ground, and the influence of soil temperature on the decomposition and accumulation of organic matter in the soil [46]. Changes in SOC and TN in the soil layer result in differences in the water stability of soil aggregates. Furthermore, the lower water content and higher porosity characteristics of tree species planted for a longer period of time than those planted for a shorter period of time lead to changes in the structure of soil aggregates, this, in turn, influences the water stability of soil aggregates. A significant negative correlation was observed between soil water content (SWC) and soil aggregate water stability, which is consistent with the findings of our study indicating that high water content is associated with lower soil aggregate water stability [47].

The findings of the study indicated that the variety and planting year of sweet cherries exerted a significant influence on the aggregate stability indexes. The Mantel test demonstrated that TN, SWC and SOC were the predominant factors influencing the aggregate stability indexes, thereby corroborating the findings of the majority of previous studies. ([48]; Lixin Wang et al., 2024). The composition of surface vegetation has a significant impact on the stability of soil aggregates. Furthermore, the distribution of soil aggregate particle sizes influences the accumulation of soil nutrients [49].

### 4.4 Effect of different varieties of sweet cherries on soil quality

The Soil Quality Index (SQI) is a comprehensive reflection of soil properties and can be used to represent soil fertility and productivity potential to some extent, Soil aggregate stability and nutrient content can be used as diagnostic indicators of SQI (M. et al., 2023; [50]). In this study, the weighting of the comprehensive analysis of the aggregate stability index and soil physicochemical properties revealed that with the increase in planting years, the quality of the soil will instead decline. The ecological benefits of apple planting on the Loess Plateau are significantly lower than those of eco-plantation. The implementation of apple cultivation has been observed to result in a decline in soil moisture, a reduction in soil aggregate stability, and an enhancement of erosion susceptibility. In light of these findings, it is recommended that caution should be exercised when planting apples in semi-arid regions, as this may have a detrimental impact on the sustainability of the region [21]. These conclusions align with those of the study. This may also be related to the neglect of scientific plantation management in large-scale plantations, where monocultures are used in orchards, which affect soil quality. Mixed cropping and intercropping may be a key tool to address the problem of declining soil quality in the future [51,52].

The results of the pathway analysis indicated that the sweet cherry variety and planting years exert a significant influence on soil quality. While planting sweet cherries in the Loess Plateau region offer economic benefits, it is also crucial to prioritize the protection of soil quality. During the planting process, the implementation of protection measures may affect soil nutrients and the stability of soil aggregates, which could potentially impede the achievement of the objective of improving soil quality.

## 5 Conclusions

The study demonstrated that the characteristics of different sweet cherry varieties affect soil aggregates stability and physicochemical factors of the soil, and then affect soil quality. It can be observed that soil quality declines with the increase in planting years. In addition, SOC and TN in the soil surface layer were the main factors affecting the stability of soil aggregates. The stability of soil aggregates exerts a significant influence on soil quality, introduction of a systematic soil aggregate stability index enables a more comprehensive evaluation of soil quality. The pursuit of economic benefits while planting fruit trees should be balanced with soil quality assessment, this study provides a scientific theoretical basis for the selection and management of sweet cherry planting on the Loess Plateau region.

## CRediT authorship contribution statement

**Muhao Chen:** Writing – original draft, Methodology, Conceptualization. **Shu Feng:** Investigation. **Jun Wang:** Writing – review & editing, Data curation. **Mingyu Gao:** Resources, Data curation. **Min Liu:** Writing – review & editing, Software. **Kaibo Wang:** Writing – review & editing, Data curation. **Zhou-ping Shangguan:** Writing – review & editing, Data curation. **Yongwang Zhang:** Supervision, Project administration, Funding acquisition, Conceptualization.

## Data availability

Data will be made available on request.

## Declaration of competing interest

The authors declare that they have no known competing financial interests or personal relationships that could have appeared to influence the work reported in this paper.
